# Drugs, sleep, and the addicted brain

**DOI:** 10.1038/s41386-019-0465-x

**Published:** 2019-07-16

**Authors:** Rita J. Valentino, Nora D. Volkow

**Affiliations:** National Institute on Drug Abuse, National Institutes of Health, 6001 Executive Blvd, Rm 4269, MSC 9555, Bethesda, MD 20892 USA

**Keywords:** Circadian rhythms and sleep, Neuroscience

The neurobiology of sleep and substance abuse interconnects, such that alterations in one process have consequences for the other. Acute exposure to drugs of abuse disrupts sleep by affecting sleep latency, duration, and quality [1]. With chronic administration, sleep disruption becomes more severe, and during abstinence, insomnia with a negative effect prevails, which drives drug craving and contributes to impulsivity and relapse. Sleep impairments associated with drug abuse also contribute to cognitive dysfunction in addicted individuals. Further, because sleep is important in memory consolidation and the process of extinction, sleep dysfunction might interfere with the learning of non-reinforced drug associations needed for recovery. Notably, current medication therapies for opioid, alcohol, or nicotine addiction do not reverse sleep dysfunctions, and this may be an obstacle to recovery [2, 3]. Whereas exposure to drugs of abuse is causal to sleep dysfunctions that further promote chronic use, sleep disorders in turn are risk factors for substance abuse and their severity can predict the prognosis of substance use disorders (SUD) [4]. Sleep disruption results in a cumulation of risk factors that drive drug abuse, including increasing the sensitivity to pain, acting as a stressor, and biasing toward a negative effect. Recognizing and treating sleep disorders may be an important preventive measure against future drug misuse and SUD. Despite convergent evidence linking sleep and substance abuse, and the therapeutic potential that can emerge from elucidating the biology underlying this link, this has been a relatively neglected area of research. A first step in advancing this area is to identify how the circuits and substrates that regulate sleep and arousal intersect with those that mediate reward and also how they are targeted by drugs of abuse.

The locus coeruleus (LC)–norepinephrine (NE) system is a diffuse forebrain-projecting system that is involved in arousal and also is a primary target of drugs of abuse, including nicotine, stimulants, opioids, and cannabinoids. LC–NE neuronal activity is positively correlated to the state of arousal, and LC neurons are most active during waking and are off during REM sleep [5]. Selective LC activation is sufficient to elicit cortical arousal, and conversely, selective LC inhibition prevents cortical activation by stressors, indicating that this system is important in regulating cortical arousal in response to stressors and other salient stimuli [6, 7]. The stress-related neuropeptide, corticotropin-releasing factor (CRF), mediates stress-induced LC excitation, and endogenous opioids that innervate the LC exert an opposing effect that may serve to restrain excessive activation and promote recovery after stress termination [8]. Opioid tolerance would be expected to enhance stress-induced activation of this arousal system, and promote a cycle of drug seeking to tone down the excessive response. LC neurons are robustly activated during opioid withdrawal and this has implicated the LC–NE system in opioid-withdrawal signs, including the hyperarousal and insomnia associated with withdrawal [9]. Notably, α2-adrenergic antagonists (lofexidine and clonidine) that inhibit LC discharge are clinically used for the attenuation of opioid and alcohol withdrawal to reduce peripheral symptoms from sympathetic activation, such as tachycardia, as well as central symptoms, such as insomnia, anxiety, and restlessness. Their utility in suppressing symptoms during protracted abstinence, such as insomnia, along with its associated adverse consequences (irritability, fatigue, dysphoria, and cognitive impairments) remains unexplored.

Like LC–NE neurons, the raphe nuclei (including the dorsal raphe nucleus—DRN) serotonin (5-HT) neurons modulate sleep and wakefulness through widespread forebrain projections. The role of this system in sleep is complex. Raphe nucleus lesions trigger insomnia [10, 11], and during the awake state, the cumulative 5-HT, released from the raphe into the basal forebrain (including the nucleus basalis, which is the main cholinergic input to the cortex, and regulates arousal), is believed to serve as a sleep-promoting factor [11]. However, 5-HT neurons are active during waking, decrease their activity during slow-wave sleep, and cease firing during REM sleep, as is the case for LC–NE neurons [12, 13]. Notably, DRN-5-HT neurons are implicated in the arousal from sleep in response to hypercapnia [14], which is impaired during opioid-induced overdoses, and further work is required to assess how to target the serotonin system as a way to prevent opioid-induced overdoses or to improve outcomes when naloxone cannot completely reverse them (Table 1).

**Table 1:** Predominant effects of neurotransmitter targets of various drugs in sleep and arousal and their typical effects during intoxication and withdrawal

Neurotransmitter	Drug	Intoxication	Abstinence
**NE** Arousing	Stimulants Opioids Alcohol	Enhanced Reduced Reduced	Reduced during early stages of withdrawal Hyperexcitable Hyperexcitable
**5-HT** Arousing/sedating	Stimulants Ecstasy	Enhanced Enhanced Enhanced	Reduced
**DA** Arousing	Stimulants Opioids Nicotine Cannabis Alcohol	All drugs enhance DA	D2R, DAT, and DA release are downregulated
**Histamine** Arousing	Opioids Alcohol	Enhanced Reduced	
**Nicotine** Arousing	Nicotine	Enhanced	Tolerance
**Orexin** Arousing	Cocaine Opioids	Enhanced Enhanced	Upregulated Upregulated
**Mu opioids** Sedating	Opioids Nicotine Alcohol	Enhanced Enhanced Enhanced	Tolerance of MOR
**Adenosine** Sedating	Caffeine	Reduced	Tolerance
**Cannabinoids** Sedating	Cannabis	Enhanced	Downregulation

Because the effects of a neurotransmitter on arousal and sleep may differ depending on the brain region it targets, in some instances, the effects are mixed as is the case for serotonin. Also, the effects can differ during early versus protracted withdrawal, such as is the case for cocaine that leads to enhanced sedation that can last up to 3–4 weeks post withdrawal to then be followed by protracted insomnia

Like the LC–NE and DRN-5-HT systems, the histamine (HA) neurons of the tuberomammillary nucleus form another diffusely projecting arousal system that is active during waking only and these neurons are activated by opioids, which can further contribute to sleep disruption associated with chronic opioid use. HA promotes arousal through activation of cortical and basal forebrain neurons, effects that are primarily mediated by H1 receptors [15, 16]. Thus, the H1 receptor may be an alternate target for treating sleep dysfunction associated with abstinence.

In contrast to the LC–NE and DRN-5-HT neurons, midbrain dopamine (DA) neurons were not considered to be sleep-related, because they show little change in discharge rate during the sleep/wake cycle other than bursting during paradoxical sleep. However, the wake-promoting actions of drugs that enhance DA signaling are widely recognized and used for clinical purposes [17, 18]. Transgenic modifications that enhance DA neurotransmission in mice, such as deletion of the DA transporter gene, result in increased wakefulness [19], whereas deletion of DA D2 receptors (D2R) decreases wakefulness [20]. Further, recent optogenetic studies demonstrated that activation of DA neurons in the ventral tegmental area (VTA) but not substantia nigra increases wakefulness [21]. These arousal effects are mediated by VTA projections to the nucleus accumbens, because optogenetic activation of DA terminals here, but not in other terminal regions, also promoted wakefulness. Therefore, this specific DA circuit is a node that regulates the rewarding effects of drugs of abuse and one that mediates arousal, including that elicited by salient and rewarding stimuli.

The endogenous cannabinoid system (ECS) that signals through cannabinoid CB1 and CB2 receptors, which are targets of marijuana, is also involved in circadian rhythm and the regulation of the sleep–wake cycle [22, 23]. Acutely, cannabis is sleep-promoting and decreases latency, increases sleep time, increases slow-wave sleep, and decreases REMs [1, 23]. Consistent with this, CB1 antagonists increase wakefulness and decrease slow-wave sleep and REMs and conversely, the endogenous CB1 agonist, anandamide enhances slow-wave sleep and REM [24]. The effects of CB1 signaling may be mediated through the sleep-promoting molecule, adenosine because doses of anandamide that promote sleep increase adenosine release in the basal forebrain [25]. Notably, adenosine is the target of caffeine, which is an adenosine receptor antagonist that is widely used to increase arousal. Another potential mechanism for the sleep-promoting effects of endocannabinoids is through their opposing regulation of neuronal activity in the lateral hypothalamus, inhibiting the activity of arousal-promoting orexin neurons (see below), while increasing the activity of sleep-promoting melanin concentrating hormone neurons [26].

With chronic use, tolerance occurs to the sleep-enhancing effects of cannabis, and abstinence is characterized by unusual dreams and poor sleep quality that is predictive of relapse [27]. ECS disruption with chronic marijuana use is likely to underlie the long-lasting insomnia commonly observed during abstinence in cannabis abusers.

The orexin system that derives from the posterior lateral hypothalamus is like the LC–NE, DRN-5-HT, and TMN–HA systems in that the cells only fire during waking and are silent during sleep phases [28]. It is unique in being essential for sustaining the waking state, as its disruption in patients with narcolepsy leads to periodic and abrupt interruptions of the conscious state. The cluster of orexin neurons in the hypothalamus is a node that links to the other arousal-related nuclei, including basal forebrain cholinergic neurons, TMN–HA neurons, DRN-5-HT neurons, VTA–DA neurons, lateral dorsal tegmental cholinergic neurons, and LC–NE neurons (Fig. 1). It is poised to orchestrate a synchronous activation of multiple arousal systems. In addition to its role in arousal, orexin has a role in the rewarding effects of drugs of abuse, including those of opioids [29]. For example, narcoleptics that have low orexin levels do not abuse opioids and mice with genetic deletion of orexin show decreased opioid-addiction potential, implicating orexin in the initial rewarding effects of opioids [29]. Orexin neurons are activated by reward and project to the DA neurons in VTA that innervate the nucleus accumbens and mediate reward, which they also influence via their direct projections to it. Chronic opioid exposure upregulates orexin in humans and rodents [30]. Postmortem brains of heroin users showed increases in orexin neuron numbers in the lateral hypothalamus and reverse translation studies verified that chronic opioid administration in rodents also increased the number of orexin neurons in their lateral hypothalamus. The upregulation of orexin would be expected to create a state of hyperarousal and may underlie the insomnia observed both in treated and non-treated opioid users. Preclinical studies, demonstrating that orexin microinjection into the VTA increases cocaine self-administration and reinstates cocaine-conditioned place preference, also implicate orexin in cocaine’s rewarding effects. It has been suggested that orexin is specifically engaged in substance abuse during elevated motivational states, such as when the effort to obtain the drug is high [29] or when animals are stressed [31]. For example, orexin antagonists only affect self-administration under conditions that require a relatively high effort, such as progressive ratio schedules, or when drug seeking is triggered by cues or stress, suggesting that it may be particularly active during relapse. This would be consistent with a state of heightened arousal that accompanies craving. These findings provide a rationale for the evaluation of orexin antagonists, such as suvorexant, a drug currently approved for use for insomnia, as therapy for substance use disorders and for the development of new ones. These agents may provide a two-fold benefit by preventing two distinct but interrelated effects of orexin, potentiation of reward and arousal effects, which could help attenuate drug reward and improve sleep disturbances.

**Fig. 1:**
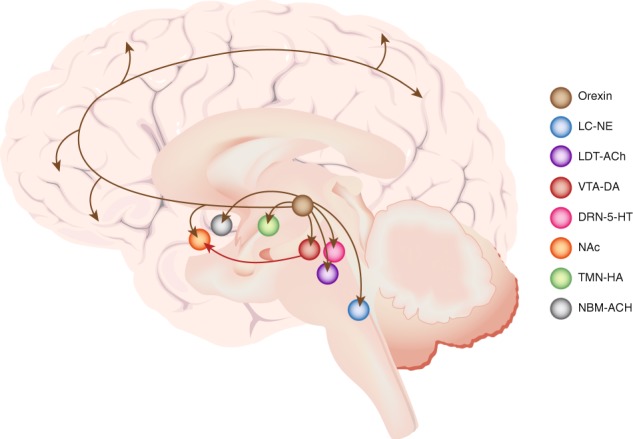
Schematic depicting efferent projections of lateral hypothalamic orexin neurons. The orexin system is positioned to influence cognitive function, arousal, and reward. Orexin neurons have broad forebrain projections. Cortical projections may modulate cognitive aspects of substance use behavior such as decision-making. In addition, they project to arousal-related nuclei, including the locus coeruleus (LC), which expresses norepinephrine (NE), dorsal raphe nucleus (DRN), which expresses serotonin (5-HT), lateral dorsotegmental nucleus (LDT), which expresses acetylcholine (ACh), tuberomammillary nucleus (TMN), which expresses histamine (HA), and nucleus basalis of Meynert (NBM), which expresses ACh. These nuclei in turn have diffuse projections throughout the forebrain. Orexin neuronal projections to the ventral tegmental area (VTA) and nucleus accumbens (NAc) are poised to modulate reward and to make rewarding stimuli arousing

Although it is becoming well accepted that there are neurobiological links between sleep dysfunction and substance abuse behavior that result in comorbidity, research is still in its infancy. Earlier, we discussed a simplified anatomical framework that identifies some of the relevant links for this comorbidity, but there are likely other pathways and substrates, some of which still need to be discovered. The precise functional interactions between these different regions and their involvement in the trajectory of drug use has not been explored in any depth. Likewise, how the interaction between sleep and substance use is shaped by genetics, life events, sex, and circadian rhythms remains unknown. Further research to fill the knowledge gaps at the intersection of sleep, drug reward, and addiction will help identify treatment targets that improve the quality of life of individuals suffering from sleep and substance use disorders.

## Funding and disclosure

This work was supported by the National Institute on Drug Abuse.

## References

[1] Angarita GA, Emadi N, Hodges S, Morgan PT (2016). Sleep abnormalities associated with alcohol, cannabis, cocaine, and opiate use: a comprehensive review. Addict Sci Clin Pr.

[2] Dunn KE, Finan PH, Andrew Tompkins D, Strain EC (2018). Frequency and correlates of sleep disturbance in methadone and buprenorphine-maintained patients. Addict Behav.

[3] Ashare RL, Lerman C, Tyndale RF, Hawk LW, George TP, Cinciripini P (2017). Sleep disturbance during smoking cessation: withdrawal or side effect of treatment?. J Smok Cessat.

[4] Dolsen MR, Harvey AG (2017). Life-time history of insomnia and hypersomnia symptoms as correlates of alcohol, cocaine and heroin use and relapse among adults seeking substance use treatment in the United States from 1991 to 1994. Addiction.

[5] Aston-Jones G, Bloom FE (1981). Activity of norepinephrine-containing locus coeruleus neurons in behaving rats anticipates fluctuations in the sleep-waking cycle. J Neurosci.

[6] Page ME, Berridge CW, Foote SL, Valentino RJ (1993). Corticotropin-releasing factor in the locus coeruleus mediates EEG activation associated with hypotensive stress. Neurosci Lett.

[7] Berridge CW, Foote SL (1991). Effects of locus coeruleus activation on electroencephalographic activity in neocortex and hippocampus. J Neurosci.

[8] Van Bockstaele EJ, Reyes BA, Valentino RJ (2010). The locus coeruleus: a key nucleus where stress and opioids intersect to mediate vulnerability to opiate abuse. Brain Res.

[9] Aghajanian GK (1978). Tolerance of locus coeruleus neurones to morphine and suppression of withdrawal response by clonidine. Nature.

[10] Dugovic C (2001). Role of serotonin in sleep mechanisms. Rev Neurol.

[11] Monti JM (2011). Serotonin control of sleep-wake behavior. Sleep Med Rev.

[12] Jacobs BL, Fornal CA (1991). Activity of brain serotonergic neurons in the behaving animal. Pharm Rev.

[13] McGinty DJ, Harper RM (1976). Dorsal raphe neurons: depression of firing during sleep in cats. Brain Res.

[14] Smith HR, Leibold NK, Rappoport DA, Ginapp CM, Purnell BS, Bode NM (2018). Dorsal raphe serotonin neurons mediate CO_2_-induced arousal from sleep. J Neurosci.

[15] Khateb A, Fort P, Pegna A, Jones BE, Muhlethaler M (1995). Cholinergic nucleus basalis neurons are excited by histamine in vitro. Neuroscience.

[16] Reiner PB, Kamondi A (1994). Mechanisms of antihistamine-induced sedation in the human brain: H1 receptor activation reduces a background leakage potassium current. Neuroscience.

[17] Killgore WD, Rupp TL, Grugle NL, Reichardt RM, Lipizzi EL, Balkin TJ (2008). Effects of dextroamphetamine, caffeine and modafinil on psychomotor vigilance test performance after 44 h of continuous wakefulness. J Sleep Res.

[18] Solt K, Cotten JF, Cimenser A, Wong KF, Chemali JJ, Brown EN (2011). Methylphenidate actively induces emergence from general anesthesia. Anesthesiology.

[19] Wisor JP, Nishino S, Sora I, Uhl GH, Mignot E, Edgar DM (2001). Dopaminergic role in stimulant-induced wakefulness. J Neurosci.

[20] Qu WM, Xu XH, Yan MM, Wang YQ, Urade Y, Huang ZL (2010). Essential role of dopamine D2 receptor in the maintenance of wakefulness, but not in homeostatic regulation of sleep, in mice. J Neurosci.

[21] Oishi Y, Lazarus M (2017). The control of sleep and wakefulness by mesolimbic dopamine systems. Neurosci Res.

[22] Vaughn LK, Denning G, Stuhr KL, de Wit H, Hill MN, Hillard CJ (2010). Endocannabinoid signalling: has it got rhythm?. Br J Pharm.

[23] Babson KA, Sottile J, Morabito D (2017). Cannabis, cannabinoids, and sleep: a review of the literature. Curr Psychiatry Rep.

[24] Murillo-Rodriguez E (2008). The role of the CB1 receptor in the regulation of sleep. Prog Neuropsychopharmacol Biol Psychiatry.

[25] Murillo-Rodriguez E, Blanco-Centurion C, Sanchez C, Piomelli D, Shiromani PJ (2003). Anandamide enhances extracellular levels of adenosine and induces sleep: an in vivo microdialysis study. Sleep.

[26] Huang H, Acuna-Goycolea C, Li Y, Cheng HM, Obrietan K, van den Pol AN (2007). Cannabinoids excite hypothalamic melanin-concentrating hormone but inhibit hypocretin/orexin neurons: implications for cannabinoid actions on food intake and cognitive arousal. J Neurosci.

[27] Babson KA, Boden MT, Harris AH, Stickle TR, Bonn-Miller MO (2013). Poor sleep quality as a risk factor for lapse following a cannabis quit attempt. J Subst Abus Treat.

[28] Jones BE (2011). Neurobiology of waking and sleeping. Handb Clin Neurol.

[29] James MH, Mahler SV, Moorman DE, Aston-Jones G (2017). A decade of orexin/hypocretin and addiction: where are we now?. Curr Top Behav Neurosci.

[30] Thannickal TC, John J, Shan L, Swaab DF, Wu MF, Ramanathan L (2018). Opiates increase the number of hypocretin-producing cells in human and mouse brain and reverse cataplexy in a mouse model of narcolepsy. Sci Transl Med.

[31] Tung LW, Lu GL, Lee YH, Yu L, Lee HJ, Leishman E (2016). Orexins contribute to restraint stress-induced cocaine relapse by endocannabinoid-mediated disinhibition of dopaminergic neurons. Nat Commun.

